# Commentary on: Subgroup analysis and interpretation for phase 3 confirmatory trials: White Paper of the EFSPI/PSI working group on subgroup analysis by Dane, Spencer, Rosenkranz, Lipkovich, and Parke

**DOI:** 10.1002/pst.1935

**Published:** 2019-03-21

**Authors:** Robert Hemmings, Armin Koch

**Affiliations:** ^1^ Medicines and Healthcare Products Regulatory Agency London UK; ^2^ Institut fur Biometrie Medizinische Hochschule Hannover Hannover Germany

Activity in journals and at conferences on the topic of subgroup analyses in clinical trials appears to be on the increase, co‐incident with, perhaps even influenced by, the release of the draft EMA guideline on the topic.^1^ The topic has always been important, difficult, and controversial, and the additional attention is welcome.

Clinicians are trained to think that patients even with the same disease belong to certain risk groups, defined by, e.g., age, disease severity or stage, or by genomic classification, where the effect of a treatment and even the preferred treatment option might differ. Biostatisticians have struggled to translate this thinking into the design and analysis of clinical trials. Indeed, a regulatory guideline that demands a close inspection of subgroups seems to be in some contradiction to biostatistical traditions in drug development that mandate a clear pre‐specification of all elements of the analysis that support confirmatory conclusions from the trial.

Biostatisticians have, for a long time, criticised those who look into subgroup analyses outside of a confirmatory testing strategy in an attempt to make something out of effects seen. Both, more favourable and less favourable subgroup findings became the focus of attention because there is a tendency to give more weight to the apparently positive result in a subgroup of a failed trial than to the apparently neutral or negative finding in a subgroup of a positive trial. In instances from a statistical perspective, the same amount of evidence was available, and only the sign of the treatment effect was reversed.

Such apparent differences in interpretation seem to mandate a strict view on the assessment of subgroups particularly as the theoretical argument standing behind the aforementioned criticism is universally understood: Testing of multiple subgroups (or endpoints) from the same trial will increase the risk of having one or more misleading findings, e.g., the risk of false‐positive conclusion about efficacy of the experimental drug. This view is well‐founded in the context of providing (and assessing) a formal proof of efficacy for a new medicine. What should then be different if we focus on an apparently neutral or even negative outcome in a subgroup of a positive trial?

In line with the usual approach of clinicians to group patients of similar risk or with similar potential for response to treatment, it is entirely plausible that the magnitude of the treatment effect will differ, at least to some extent, between well‐defined subgroups. There might be established knowledge about the prognostic value of factors like gender or disease stage, level of biomarker expression, or concomitant medication, and these or other factors might also be predictive for treatment outcome. This is to be expected and does not necessarily introduce problems. Following formal proof of efficacy, regulatory assessment needs to address not only the balance of benefits and risks of treatment for the population as a whole but also whether the treatment effects estimated are broadly consistent across the full patient population. Where beneficial effects are truly inadequate, or risk‐benefit is truly unfavourable in a well‐defined subpopulation, these patients should not be treated. Therefore, the assessment of subgroups is an integral part of risk‐benefit assessment even if it is plausible, and is the basis for planning the trial, that the new drug should benefit all parts of the target patient population. Given that the current paradigm of phase 3 clinical research is still to treat a broad and at best unrestricted target population with a certain disease, it seems appropriate that any a priori assumption of similar efficacy across the target population is checked after the trial has been completed. We do not pretend that this is an easy task.

It should be acknowledged that clinical trial data permits investigation of subgroups based on a well‐understood and well‐documented database, in which randomised groups of patients can be compared. Nevertheless, these investigations must proceed with caution, and the analysis of subgroups presents a problem that will always be somewhat intractable: multiple questions can be posed, each to be addressed with limited information in trials that were planned to support a confirmatory conclusion in the whole trial population.

The authors of the White Paper reflect on current practice, based largely on interaction tests in somewhat complex regression models, unadjusted analyses of individual subgroups, and presentation of findings in Forest plots. We can agree that such methods are rather basic. In particular, Forest plots are univariate and present findings with opaque correlations between different subgroups, rendering the interpretation of “consistency” difficult. Tests for interaction are of principal interest, but lack adequate sensitivity and specificity to detect or to exclude truly differential efficacy in the study population and so are considered to be a crude tool with well‐understood limitations. The dichotomous decision about presence or absence of an interaction on the basis of a statistical test, with arbitrary criteria for “success,” cannot be promoted as a reliable basis for decision making in regulatory guidance or the effects seen in trials under review.

The authors refer to the draft EMA guideline on investigation of subgroups, the final version of which is to be published early in 2019. This document aims to provide a framework for consideration of subgroups in regulatory decision making with implications for trial planning, analysis, inference, and interpretation. In order not to become restrictive or quickly outdated, the guideline, in line with all EMA methodological guidance documents, does not seek to prioritise or impose any particular methodological approach but describes the information needed to support decision making. Of course, research leading to useful advancement from basic methodology is welcome.

The guideline promotes the idea that methodological investigations alone cannot be definitive for decision making. The focus of the guideline is to use statistical methods for signal generation, specifically to identify subgroups in which treatment effects might differ. These findings can then be interpreted for their credibility and relevance. One suggestion described in the draft EMA guideline is to place investigation of subgroups in a hierarchy. This idea is mirrored in the White Paper. Where inconsistency of effects in subpopulations is highly plausible, the conduct of separate studies in the respective subpopulations may be the most efficient research strategy. Where there is no basis to consider strong inconsistency of effects between particular subgroups plausible, subgroup‐analyses will be planned outside the confirmatory testing strategy and will be analysed to investigate the internal consistency of the trial results in a check that the overall treatment effect applies to relevant subpopulations of the patient population. However, even in this case, there will be factors for which some biological plausibility or external evidence exist that response to treatment might not be consistent. It is disingenuous to suppose otherwise. Examples might include factors used to stratify randomisation, key demographic or disease‐related factors, including genomic factors, and factors related to the mechanism of action. These factors and the subgroups based thereon merit additional attention over subgroups where consistent treatment effects can be expected.

The extent of population heterogeneity and potential inconsistency in treatment effects should be considered at the planning stage. This is not trivial, since even after an extensive exploratory development programme, information is limited, and it is undesirable to restrict patient populations in clinical development so much that external validity is compromised. Planning should extend to considerations of stratification and overall sample size. Indeed, strong prognostic factors have always been used for stratification of the randomisation. This not only is to balance (in combination with blocking randomisation) the treatment groups in respect of the patients' prognosis and to increase efficiency of statistical tests but also indicates an intention to compare effects across important subpopulations. Comparison might address, for example, the question whether patients with low risk achieve the same treatment effect as high risk patients. Stratified randomisation creates a trial within a trial and indicates that a pre‐specified comparison of like with like is of interest during assessment. Obviously, for all treatment comparisons, it is important to check whether treatment groups are balanced with respect to (other) important prognostic factors. If not, it is important to utilise all techniques according to best epidemiological practice to adjust for differences in baseline that may impact on the treatment effect of interest in sensitivity analyses. Ignoring apparent baseline imbalances is compatible with controlling a pre‐specified type 1 error in decision making on the long run, but is unwise for motivating a positive decision against better knowledge (e.g., if despite randomisation a baseline‐imbalance in an important prognostic factor gives a clear disadvantage to the placebo‐group).

The categorisation of factors according to their importance is introduced to give a priori weight to the importance of subgroup findings in the subgroups created by the many available baseline characteristics. Discussed at the planning stage, this prioritisation is a means to make the heterogeneity of the patient population transparent to the readership of the clinical trial and is one mechanism to reduce the attention given to eye‐catching and potentially misleading findings (subgroup‐findings that appear different to the overall effect). All interested parties should contribute to the respective discussion in the study protocol with their knowledge about the plausibility for inconsistency in treatment effects, evidence for a class‐effect, or other medical or epidemiological evidence. Admittedly, the possibility to reduce the number of subgroups to be inspected is limited by the need to describe the patient population in the trial and thus cannot be arbitrarily fixed to a certain minimal number. This comprehensive description presents a challenge for the proper planning of a clinical trial and is a potential topic for discussions when engaging in Scientific Advice.

The potential for one or more factors to define subgroups where the effect of treatment is truly different should not be dismissed. Because authorisation of a medicine depends on demonstration of therapeutic efficacy, including considerations of clinical relevance, and the trade‐off between the extent of benefits and the profile of risks, difference in the effect of treatment that could be important for decision making relate to the magnitude of the effects, and not only whether effects are consistent in direction. Even if most factors defining subgroups of potential interest are unlikely to be informative in determining response to treatment, in our experience, there will be some in each trial where important inconsistency cannot be dismissed a priori. If, once the trial data are available, a lower treatment effect is estimated in a subgroup, and this finding is plausible, it is hard to argue in the context of a regulatory assessment that this should be simply dismissed without further investigation and assessment as “a chance finding in one of multiple subgroups.” For example, consider a new treatment concept being compared (perhaps a targeted therapy in oncology) to an existing treatment strategy. Non‐inferiority might be an acceptable objective for the trial, but subgroups may be defined (or exist still undetected), where the old drug may be worse (because the new mechanism of action truly leads to an improvement in a targeted subgroup). For the same reason, the old drug may be the better treatment approach for some subgroups, and this then should be acknowledged, as well. For obvious reasons, this discussion cannot be completed at the planning stage.

Statisticians can help in various aspects. We appreciate the initiative of colleagues of the EFSPI/PSI working group to develop methodology to assess the strength of a signal, or the likelihood that a signal is a chance finding, or to provide descriptive methods to better understand the type of signal seen. All these are very welcome and needed in providing a thorough discussion of clinical trial results.

Three methods are proposed in the paper under an overall claim to “aid interpretation and provide context for the observed results for subgroup analyses ….” This proportionate claim is welcome. A stronger claim that any one approach, or a combination thereof, should rather be regarded as definitive would be difficult to support. Also welcome is that the choice of method will depend on the aim of the work: to provide context, to help understand the correlation structure in the data, to provide adjusted estimates, or to provide adjusted *P* values. The draft EMA guideline focusses on investigation of subgroups in decision making, but regulators also need to describe effects in subgroups in product labelling or public assessment reports. The distinction between inference and description could become important in the acceptance of different methods.

Further, the standardised effect plot is a welcome addition to the panel of available analytical approaches and data presentations. Dichotomisation is required, and this represents a limitation, though we note that dichotomisation can anyway be used, or even required, for some product labelling (e.g., in the event of a restriction to the target population). The plot might have broad application since it is based on the trial data itself without an assumption that consistency of effects is more or less likely. It is noted that the performance of the plot is characterised in terms of “controlling the chances of incorrectly identifying a subgroup when there is no subgroup heterogeneity at P <10%.” Performance in terms of the likelihood of identifying a true difference would also be interesting to understand: regulatory assessment needs to identify subgroups with reduced efficacy, where re‐assurance for a still positive risk‐benefit may be needed.

Bootstrap‐adjustment seeks to address the reported fact that (point) estimates for treatment effects within subgroups are exaggerated. This is true on average where subgroups are identified in a data‐driven manner, but is not necessarily true for any individual result and should be less of a problem if an improved framework is implemented where factors and subgroups with some plausibility for differential treatment effects are targeted in advance. Bootstrap adjustment and the Bayesian shrinkage approaches that are propagated elsewhere could be of some use when needing to describe effects in (relatively small) subgroups, where there is no a priori reason to believe that the treatment effect will be inconsistent. A broader use is less attractive. Specifically, an analysis or data presentation predicated on the assumption that treatment effects are consistent seems inappropriate for a signal generation exercise. Regulators are supposed to critically challenge the treatment recommendation for a new drug. Identifying (or excluding the existence of) important inconsistencies in treatment effects across important subgroups of the target population is one way of doing this. The a priori discussion about the plausibility of negative effects expected in subgroups of the patient population depends on current knowledge at the planning stage and may justify restrictions in criteria for inclusion and exclusion. After the fact, a regulator's first interest is to use the trial data to increase knowledge about the appropriateness of the selected patient population and hence to improve the basis for decision making by discussing factors that may impact the risk‐benefit assessment. Of course, there is one important role for shrinkage‐estimates for subgroup findings that all stakeholders might support: If positive effects estimated in a subgroup analysis of a first trial are supposed to be replicated, it is extremely relevant to avoid over‐optimistic assumptions for sample‐size calculation, because the replication trial may fail if planned based on a random high finding in the subgroup.

Unfortunately, the current discussion about the relevance of subgroup findings amongst methodologists rarely gets past the question as to whether differential effects seen in subgroups can ever be more than a chance finding. This inertia might prevent cautious exploration of subgroups and eventually the possibility to learn something that may improve drug treatment. Multiple examples have been published that clarify the importance of subgroup findings in this aspect: PLATO and the combination of ticagrelor with high‐dose aspirin, the IPASS‐trial and the importance of the mutation status for treatment with gefitinib, or K‐RAS status for panitumumab are only three out of a large series of exemplar cases.

For this reason, we tend to disagree with the proposed approach in the White Paper that in the field of exploratory analyses control of type‐1‐error needs to be exercised through statistical methods. Rather, improved methods might better support signal generation and intelligent assessment. Arguably, power should be prioritised over Type I error where the objective is to generate signals for further inspection. Whilst sponsors might fear that this will lead to regulators over‐interpreting results from one of multiple subgroup analyses, the guideline outlines that any signal will be assessed for its credibility considering whether it is replicated in other relevant data sources or has biological plausibility. These careful considerations mitigate the risk for regulatory action based on the trial data alone. It is important to remember that assessment of subgroups is a two‐sided problem: just as regulatory action based on a false negative is undesirable, so is failure to identify a true negative result from a subgroup, indicating lower effect or harm in a subgroup of the patient population. When the White Paper discusses (in the context of classical interaction tests) only the risks of increased type 1 error and lack of power, the latter is (for the regulatory assessment) the more important aspect. So, in essence, four things are important:

1. There are no alternatives to a careful inspection of subgroups and subgroup findings in clinical trials
2. The plausibility of signals found in this assessment needs to be discussed from a medical and biological perspective. If done properly at the planning stage, an a priori discussion can mitigate the risk of multiple subgroups' leading to false‐positive results.
3. After carefully assessing the plausibility of the finding, a discussion may be appropriate and helpful, of how likely a signal is “just a chance finding.” Here, all proposals for intelligent methodological approaches that may shed light on a difficult problem are welcome.
4. Both, regulators and sponsors need to change their attitudes: in the current discussion, “a signal” is mainly seen as a threat to a development program. Instead, a signal should be understood as an opportunity to learn in how far an average treatment effect applies to the whole patient population under investigation. The clinical trial is a model answering the question about what would have happened if a certain patient had been treated with the experimental treatment instead of control. Subgroup analyses are a means to explore whether average treatment effects identified apply across the breadth of the target population. Information from subgroup analyses can then be considered judiciously in regulatory opinions, in full awareness that results of these analyses can be misleading when considered without the context provided by other relevant data and expert insights from clinical pharmacologists and medical doctors.

Doubtless further research will extend and optimise the different methods proposed, and the different approaches described might each find a different role in the exploration of subgroups, but the development and presentation of the methods is an extremely welcome reaction to the draft guideline. The members of the PSI/EFSPI working groups are to be congratulated and commended for the work done to date. There are a number of well‐known examples where, after careful and difficult reflection, decisions have been made to exclude subgroups from the approved Therapeutic Indication. This has sometimes been motivated in reaction to safety findings, but in other instances by a need to restrict the target population in order to achieve a favourable risk‐benefit.

A plausible next step for the EFSPI/PSI working group should be to investigate how the methods proposed perform when applied to such data sets and to discuss to what extent the proposals outlined here lead to results that are consistent with, or in contradiction to, the conclusions that were reached in those regulatory decisions.
